# Foodborne Pathogens Across Different Food Matrices in Sicily (Southern Italy)

**DOI:** 10.3390/pathogens13110998

**Published:** 2024-11-14

**Authors:** Valeria Blanda, Ilenia Giacchino, Valeria Vaglica, Vanessa Milioto, Sergio Migliore, Santina Di Bella, Francesca Gucciardi, Carmelo Bongiorno, Giuseppina Chiarenza, Cinzia Cardamone, Isabella Mancuso, Maria Luisa Scatassa, Vincenza Cannella, Annalisa Guercio, Giuseppa Purpari, Francesca Grippi

**Affiliations:** Istituto Zooprofilattico Sperimentale della Sicilia A. Mirri, Via G. Marinuzzi 3, 90129 Palermo, Italy; valeria.blanda@izssicilia.it (V.B.); giacchino.ilenia@gmail.com (I.G.); vaglicavaleria@gmail.com (V.V.); vane.milioto89@gmail.com (V.M.); sergio.migliore@izssicilia.it (S.M.); carmelo.bongiorno@izssicilia.it (C.B.); giuseppina.chiarenza@izssicilia.it (G.C.); cinzia.cardamone@izssicilia.it (C.C.); isabella.mancuso@izssicilia.it (I.M.); luisa.scatassa@izssicilia.it (M.L.S.); vincenza.cannella@izssicilia.it (V.C.); annalisa.guercio@izssicilia.it (A.G.); giuseppa.purpari@izssicilia.it (G.P.); francesca.grippi@izssicilia.it (F.G.)

**Keywords:** foodborne pathogens, viruses, bacteria, *Toxoplasma gondii*, zoonosis, milk, meat, mussels, vegetables, game meat

## Abstract

Foodborne diseases result from the consumption of foods contaminated with pathogens or their toxins and represent a serious public health problem worldwide. This study aimed to assess the presence of Rotavirus (RoV), Adenovirus (AdV), Norovirus (NoV), Hepatitis A and Hepatitis E viruses (HAV and HEV, respectively), *Toxoplasma gondii*, *Coxiella burnetii* and *Leptospira* spp. across various food matrices in Sicily. The analysis concerned 504 samples, including mussels, farmed meat, game meat, vegetables and bulk milk. Following appropriate pre-treatment, acid nucleic extraction was carried out and amplification of pathogen nucleic acids was carried out by molecular methods. The mussels tested positive for NoVs (3/51, 5.9%) and farm meat resulted positive for *T. gondii* (1/34, 2.9%). The game offal samples tested positive for HEV, which was detected in 17 out of 222 samples (7.7%), and *T. gondii* (18/318, 5.7%) and *Leptospira* spp. (2/318, 0.6%). The milk samples tested positive for *C. burnetii* (15/85, 17.6%), *T. gondii* (2/85, 2.4%) and *Leptospira* spp. (1/85, 1.2%). This study highlights the variability in the risk of contamination of different food matrices, confirming the importance of vigilance in the consumption of potentially contaminated food products.

## 1. Introduction

Foodborne diseases (FBDs) result from the consumption of foods contaminated with pathogens or their toxins and represent a serious public health problem worldwide. They are the leading cause of morbidity and mortality in low-income countries, while in high-income countries, they represent a significant morbidity issue and place a considerable burden on healthcare expenditure [1,2]. In Europe, the number of reported foodborne outbreaks in 2022 was 1.3 per 100,000 people, and the number of foodborne cases was 10.8 per 100,000. These values were higher than those reported between 2018 and 2021, when the rates were 0.97 outbreaks and eight cases per 100,000 people, respectively [3].

Enteric viruses of human and animal origin are widely present in the environment and are among the most frequently detected causative agents in foodborne outbreaks, posing a significant global public health risk [4]. The World Health Organization (WHO) identifies bivalve mollusks, vegetables and processed foods as priority hazards for viral contamination, as these foods are particularly susceptible to exposure [5].

In Europe, Norovirus (NoV) accounted for the highest number of human cases (N = 7305) in 2022, associated with numerous large-scale outbreaks. These outbreaks were linked to a wide variety of food items, including bovine meat, buffet meals, cheese and dairy products, mixed foods, vegetables, juices, and related products and water. Other viruses reported to have caused foodborne outbreaks in 2022 included Hepatitis A (HAV), Hepatitis E (HEV), Flavivirus, Rotavirus (RoV) [3] and Adenovirus (AdV) [6]. These viruses are primarily transmitted via the fecal–oral route. Human enteric viruses are excreted in the feces of infected individuals and can enter coastal waters through inadequately treated wastewater, sewage overflows, or direct discharges of untreated sewage. Due to their filter-feeding nature, shellfish, particularly bivalves, accumulate these viruses and are proposed as indicators of aquatic microbial contamination [7].

NoVs are non-enveloped, single-stranded RNA viruses belonging to the *Caliciviridae* family and exhibit extensive genetic variation. Human infections are primarily caused by genogroups I and II, with genotype GII.4 and its variants being especially efficient in transmission, often through food handlers. The high transmissibility of NoV is attributed to the fact that only a small infectious dose is needed to cause illness, and individuals of all ages are susceptible. While person-to-person transmission is common, recent estimates suggest that around 14% of NoV infections globally are foodborne, with approximately 59% of all foodborne illnesses linked to NoV [8]. Contamination can occur either through water containing human fecal material or, more commonly, via infected food handlers during food preparation, directly or through contaminated surfaces [9].

Adenovirus, a non-enveloped DNA virus from the *Adenoviridae* family, can cause both respiratory and gastrointestinal illnesses, and transmission via the fecal–oral route is well documented. AdV is a diverse group of viruses, with certain types, particularly AdV types 40 and 41, commonly associated with gastroenteritis, especially in children. High concentrations of AdV are often detected in sewage due to its prevalence in populations, making it a useful marker for fecal contamination in water sources [8].

Rotavirus, another major cause of gastroenteritis, particularly in children, is a double-stranded RNA virus from the *Reoviridae* family. Its triple-layered external capsid grants it substantial resistance to environmental stressors, allowing it to persist in contaminated water and food, thus facilitating its transmission [10].

Hepatitis A virus and HEV, both transmitted via the fecal–oral route, are responsible for causing acute liver infections. HAV, from the *Picornaviridae* family, is a major cause of foodborne hepatitis outbreaks, while HEV, from the *Hepeviridae* family, is increasingly associated with zoonotic transmission in Europe. In particular, HEV outbreaks have been linked to the consumption of raw or undercooked animal products, including sausages containing pig liver or wild boar meat [11].

Foodborne parasites have traditionally been under-investigated due to their complex life cycles, prolonged incubation periods, diverse transmission routes, and chronic effects on hosts. However, some zoonotic foodborne pathogens (FBPs) are increasingly recognized as emerging threats, contributing significantly to the global disease burden [12]. Common protozoan parasitic infections are frequently transmitted through food contaminated by feces-laden soil or water or by consuming meat from infected animals [2]. In Europe, *Toxoplasma gondii*, an obligate intracellular parasite with a complex life cycle, is one of the most critical foodborne parasites. The European Union One Health 2022 Zoonoses Report by EFSA states that in 2021, there were 150 confirmed cases of human toxoplasmosis, corresponding to a notification rate of 5.6 cases per 100,000 live births, marking a 10% increase compared to 2020 (5.1 cases per 100,000 live births) [EFSA]. While the parasite reproduces sexually in Felidae (its definitive host), it also infects warm-blooded animals, including humans, through contaminated water, fruits, vegetables, shellfish and undercooked or raw meats or milk. Foodborne transmission is the primary infection route for humans, with studies estimating that 30–63% of infections are linked to meat consumption, particularly that of cured and game meats [12]. The consumption of contaminated foods can also result in the transmission of bacteria such as *Coxiella burnetii* and *Leptospira* spp. The obligate intracellular bacterium *C. burnetii* has as its primary reservoir wild and domestic mammals, birds and arthropods like ticks [13]. Domestic ruminants are considered the main source of human infections. The primary mode of transmission to humans is through the inhalation of aerosols contaminated with *C. burnetii* from infected animals, particularly during parturition, when the pathogen is released in large quantities through placental tissues, milk, feces and urine [14]. *C. burnetii* can potentially be transmitted through foodborne routes and also by ticks, although the role of ticks in the transmission of the pathogen remains debated [15]. In Europe, most clinical cases are sporadic; however, several outbreaks among humans have been reported [3].

Leptospirosis is caused by infection with *Leptospira* spp. bacteria. Humans can contract leptospirosis directly through contact with the urine of infected animals or indirectly through the contamination of water and soil. Leptospirosis can have an asymptomatic course or cause fever, vomiting, hemorrhages, jaundice and kidney damage or can be lethal [16].

The aim of this study was to comprehensively evaluate the presence of a wide range of foodborne pathogens, including viruses, bacteria and protozoa, across diverse food matrices in Sicily. Specifically, the study targeted both farmed and wild game meat, bovine and ovine milk, mussels and vegetables, in order to assess potential sources of contamination and transmission risks to human health.

## 2. Materials and Methods

### 2.1. Sample Collection

The analysis included 504 samples collected during the years 2022 and 2023 in Sicily (Table 1): 85 samples of bulk milk from sheep or cattle; 34 samples of meat from farmed animals (cattle, pigs, and poultry); 51 mussel samples; 16 vegetable samples; and 318 samples derived from game meat, specifically wild boars. The samples were received in the laboratories of the Istituto Zooprofilattico Sperimentale of Sicily as part of surveillance plans or official controls. Game meat was derived from the wild boar depopulation plan activated in Sicily, following the exponential and uncontrolled increase in these animals on the island. The meat obtained from these culls has been exclusively intended for personal consumption, for example, for the preparation of cured meats and sausages, and therefore is not subject to veterinary official inspections. Consequently, no information is available regarding the potential health risks associated with the consumption of these products. Offal game meat included in the study were of the following types: spleen, liver, heart, lung, kidney and gut.

### 2.2. Sample Preparation for Protozoan and Bacterial DNA Extraction

Samples were subjected to appropriate pre-treatment protocols, as reported below.

#### 2.2.1. Meat

After flaming the surface, 1 g of sample was pre-diluted 1:10 with sterile saline solution and homogenized by the Stomacher^®^ 80 Biomaster Lab Blender (Seward Ltd., Worthing, West Sussex, UK). For DNA extraction, 200 µL of the homogenate was added with 200 µL of digestion buffer, 10 µL of Internal Control DNA (High conc.) (QIAGEN, Venlo, The Netherlands) and 20 µL of proteinase K 20 mg/mL, and incubated at 55 °C overnight. After incubation, DNA was extracted by commercial kits as reported below.

#### 2.2.2. Vegetables

Vegetable samples were pre-treated, following the protocol adopted by the European Reference Laboratory for Parasites of the Istituto Superiore di Sanità [17]. In detail, 50 g of samples was added to 200 mL of 1 M Glycine buffer (pH 5.5) and homogenized by a Stomacher^®^ 80 Biomaster Lab Blender (Seward Ltd., Worthing, West Sussex, UK). After two homogenization cycles at 300 rpm for 60 s, the homogenate was collected and centrifuged (2500× *g* at 4 °C, 10 min). Moreover, two washing cycles of the homogenizer bag were carried out with 10 mL Glycine buffer. Washing liquid was collected and centrifuged as above. Sediments obtained from the homogenized sample and from the two washing cycles of the homogenizer bag were combined in a single tube and 50 mL of Milli-Q water was added. Samples were centrifuged as above, resuspended in 900 µL of Phosphate-Buffered Saline (PBS, FastDNA Spin kit for Soil, MP Biochemicals, Santa Ana, CA, USA) added to 10 µL of Internal Control DNA (High conc.) (QIAGEN, Venlo, The Netherlands). Samples were placed in a freezer for at least 48 h before proceeding with the assay.

#### 2.2.3. Mussels

Samples of 5 g were subjected to homogenization by the Stomacher^®^ in 20 mL of 1 M Glycine buffer (pH 5.5). The homogenate was collected and centrifuged as described for vegetables. Moreover, two washing cycles of the homogenizer bag were carried out with 10 mL Glycine buffer. Washing liquid was collected and centrifuged as above. Sediments obtained from the homogenized sample and from the two washing cycles of the homogenizer bag were combined in a single tube and 15 mL of Milli-Q water was added. Samples were centrifuged and treated as described for vegetables.

#### 2.2.4. Milk

Bulk milk aliquots of 50 mL were centrifuged (2200× *g* at 4 °C, 10 min), the cream layer was removed and obtained pellets were resuspended in 200 µL of 10 mM Tris-1 mM EDTA and 300 µL of 0.5 M EDTA (Invitrogen by ThermoFisher Scientific, Waltham, MA, USA). The suspension was placed in a shaking incubator for 45 min and centrifuged (3000× *g* at 4 °C, 10 min). Supernatant was removed and pellet resuspended in 200 µL of PBS. After adding 200 µL of digestion buffer, 10 µL of Internal Control DNA (High conc.) (QIAGEN, Venlo, The Netherlands) and 20 µL of proteinase K (Invitrogen by ThermoFisher Scientific, Waltham, MA, USA), samples were incubated at 55 °C overnight in order to proceed with subsequent extraction steps according to the manufacturer’s instructions for the extraction kits.

### 2.3. Sample Preparation for Viral Genomic Extraction

#### 2.3.1. Meat

Meat samples were processed by homogenization (10% *w*/*v*) in Eagle’s Minimum Essential Medium (Sigma, St. Louis, MO, USA) supplemented with antibiotics and antimycotic agents (1000 U/mL penicillin G sodium salt, 1 mg/mL streptomycin sulfate, 2.5 μg/mL amphotericin B) (Sigma, St. Louis, MO, USA). The homogenates were centrifuged at 1500× *g* for 15 min at 4 °C. The supernatants were collected, incubated at 37 °C for 1 h, and then stored at −80 °C until further processing for biomolecular analyses.

#### 2.3.2. Vegetables

The concentration of viruses from vegetables was performed as previously detailed by Purpari and colleagues [18]. Briefly, 25 g of each sample was coarsely chopped and spiked with 10 μL of a titrated Mengovirus process control strain to assess extraction efficiency, following ISO 15216-2 guidelines [19]. The samples were homogenized in 40 mL of elution buffer (Tris/Glycine/Beef Extract, pH 9.5), agitated at room temperature, and centrifuged. The aqueous phase was collected, pH adjusted to 7.2, and polyethylene glycol (PEG) 8000/NaCl was added. After further incubation and centrifugation, the viral pellet was resuspended in PBS with antibiotics and subjected to decontamination before the upper aqueous phase containing viruses was recovered for analysis or stored at −20 °C until needed.

#### 2.3.3. Mussels

The presence of enteric viruses in mussels was evaluated following the ISO 15216-2 standard method, as previously described [20]. Briefly, at least 10 mollusks from each batch were randomly selected, and their hepatopancreases were dissected. Two grams of homogenized tissue was spiked with Mengovirus as a process control to assess extraction efficiency. The samples were digested with proteinase K, followed by centrifugation, and the supernatants were used for nucleic acid extraction.

### 2.4. Nucleic Acid Extraction

DNA was extracted from the pre-treated samples using the PureLink Genomic DNA Mini Kit (Invitrogen by ThermoFisher Scientific, Waltham, MA, USA) or QIAamp DNA Mini Kit (QIAGEN, Venlo, The Netherlands) for meat and milk samples, and FastDNA Spin Kit for Soil (MP Biochemicals, Santa Ana, CA, USA) for mussels and vegetables following the manufacturer’s instructions.

Viral RNA was extracted using the NucliSENS miniMAG extraction kit (BioMerieux, Paris, France), following the manufacturer’s instructions. The concentration and purity of the extracted nucleic acids were estimated using a NanoDrop spectrophotometer (ThermoFisher, Waltham, MA, USA).

### 2.5. Detection of FBP Nucleic Acids in Extracted Samples

*Toxoplasma gondii* DNA was detected via real-time PCR targeting the 529 bp repeat element [21]. In each reaction, positive controls, containing DNA extracted from reference cultured positive samples, and negative controls, containing sterile distilled water, were added. The real-time PCR reaction mix was composed of 1X SsoAdvanced Universal Probes Supermix (Bio Rad, Hercules, CA, USA), 500 nM of each primer and 12.5 nM of probe, 1X EXO IPC Mix and 1X EXO IPC DNA in a 20 µL total volume and the following thermal cycle conditions were used: 95 °C for 3 min, 40 cycles of 95 °C for 10 s and 60 °C for 30 s.

*Coxiella burnetii* DNA was amplified via real-time PCR targeting the IS1111 region of 16S ribosomal RNA [22]. The reaction mix included 1X SsoAdvanced Universal Probes Supermix (Bio Rad), 500 nM of each primer and 500 nM of probe, in a 20 µL total volume. Thermal cycle conditions were same as above.

For *Leptospira* spp., a real-time multiplex PCR method was used. This reaction detects all *Leptospira* species by amplifying a conserved region of the 16S rRNA gene and discriminates pathogenic species (*L. alexanderi*, *L. borgpetersenii*, *L. interrogans*, *L. kirschneri*, *L. noguchii*, *L. santarosai*, *L. weilii* and *L. astonii*) by amplifying a highly conserved region of the lipL32 gene (Lipoprotein L32), expressed only in pathogenic species [23,24]. The reaction mix was composed of 1X Mastermix Quantifast (Quiagen), 500 nM of each Lep-primer, 150 nM of Lep-probe, 700 nM of each Lip32l primers, 200 nM of Lip32l probe and 1X of Internal Control assay. The thermal cycle conditions of the real-time PCR were 95 °C for 5 min, 45 cycles of 95 °C for 15 s and 60 °C for 30 s.

All the real-time PCRs were performed on a QuantStudio™ 6 Pro Real-Time PCR System (Life Technologies, Thermo Fisher Scientific, Carlsbad, CA, USA).

Norovirus, HAV and HEV were detected using real-time reverse transcription PCR with the RNA UltraSense One-Step qRT-PCR System (Invitrogen, Thermo Fisher Scientific, Carlsbad, CA, USA) as widely described elsewhere [18,20].

For RoV amplification, prior to adding RNA to the RT-PCR master mix, sample RNA was subjected to denaturation at 97 °C for 5 min, followed by incubation on ice for 2 min to separate the viral dsRNA. Reverse transcription was carried out using Taq DNA Polymerase PCR Buffer, Random Primers and M-MLV Reverse Transcriptase (Invitrogen, Thermo Fisher Scientific, Carlsbad, CA, USA), and PCR amplification was performed using the TaqMan Universal PCR Master Mix (Applied Biosystems, Thermo Fisher Scientific, Carlsbad, CA, USA) [25].

Adenovirus detection was performed using nested PCR [20]. The primers, probes and PCR cycling parameters for each assay have been reported previously [18,20], and target regions along with references are presented in Table 1. All real-time RT-PCR assays were performed on the QuantStudio™ 6 Pro Real-Time PCR System. Each run included molecular-grade water and a positive RNA/DNA template as quality controls.

Virological analyses were conducted on more common and well-documented sources of contamination which are recognized as primary vehicles for these pathogens. Farmed meat and game offal were excluded from HAV, AdV and NoV GI/GII analysis, as these matrices are typically associated with secondary contamination, often due to improper food handling, rather than primary contamination. Milk samples were not tested for viral pathogens as the volume of the received samples was insufficient.

Details of molecular methods carried out in this study are reported in Table 2. Genbank Accession Numbers of the target regions amplified in this study are reported in Table S1.

## 3. Results

The results of the investigations carried out on the different food matrices are reported in Table 3.

Three mussels were positive for NoV (3/51, 5.9%); in particular, one sample belonged to the GI genotype and two samples belonged to the GII genotype. *Toxoplasma gondii* DNA was detected in one sample (1/34, 2.9%) of farm meat.

Regarding the game offal, *T. gondii* was detected in 18 out of 318 samples (5.7%), pathogenic *Leptospira* spp. in 2/318 (0.6%) and HEV virus in 17/222 (7.7%).

Concerning the milk samples, 15 out of 85 milk samples (17.6%) tested positive for *C. burnetii*. Of these, 4 were detected among the bovine milk samples (4/48, 8.3%) and 11 among the ovine milk samples (11/37, 29.7%). Two milk samples (2/85, 2.4%) tested positive for *T. gondii*. Both positive milk samples were of bovine origin (2/48, 4.2%). One milk sample (1/85, 1.2%), of bovine origin (1/48, 2.1%), tested positive for *Leptospira* spp.

All other analyses were negative.

## 4. Discussion

Our study aimed to comprehensively evaluate the presence of a wide range of foodborne pathogens, including viruses, bacteria and protozoa, in various food matrices in Sicily. Mussels were found to be positive for NoV, detected in 3 out of 51 samples. One sample was identified as belonging to the GI genotype, while two samples were classified under the GII genotype. In farm meat, *T. gondii* DNA was detected in one sample of minced beef. Game offal meat was detected to be positive for HEV, *T. gondii* and *Leptospira* spp. Regarding the milk samples, 15 out of 85 were positive for *C. burnetii*, detected in both the bovine and ovine milk samples. Additionally, two bovine milk samples tested positive for *T. gondii*, and one bovine milk sample was positive for *Leptospira* spp. Overall, these data report a prevalence of 4.2% for FBPs in the examined food matrices.

Norovirus was the agent associated with the highest number of human outbreak cases in 2022 [3]. NoV GI and GII are the main causes of gastroenteritis in humans affecting both children and adults. They have been detected in treated wastewater, surface waters and shellfish worldwide [32,33]. Previous outbreaks of NoV gastroenteritis in Sicily were linked to contaminated drinking water from municipal supplies in the Agrigento and Catania provinces (Sicily, Italy) [34,35]. Mussels are the primary vehicles of NoV infection due to their water-filtering activity, which leads to the accumulation of various pathogens [18,36]. Their high probability of contamination is of concern because, although NoV is easily inactivated by cooking [37], these shellfish are often consumed raw, particularly in some areas of Southern Italy and France [38]. Therefore, this habit represents a gap in the control of an otherwise easily preventable disease. The identification of NoV GII as the predominant genotype aligns with the global distribution of NoV GII in the human population [39] and with a previous survey of various food matrices in Italy reporting that 2.2% of food samples were contaminated by at least one virus type, with NoV GII being the most detected pathogen, especially in mollusks [40]. Although, in this study, the vegetable samples were all negative for NoV, the presence of this pathogen in “ready to eat” vegetables has been documented in Italy, highlighting a potential risk associated with the consumption of such products [41].

This study also reports an HEV prevalence of 7.7% in wild boars in Sicily. The presence of HEV in game offal, particularly the liver, suggests a potential risk associated with the consumption of undercooked or raw wild boar meat [11], with implications for food safety, the game meat production chain and economic aspects. HEV has become an important microbiological factor requiring increased attention from health authorities. In 2023, 58 cases of Hepatitis E were reported in Italy, with 10.2% of individuals reporting the consumption of raw or undercooked wild boar meat [42]. This study confirmed HEV circulation in wild boars in Southern Italy. The transmission of zoonotic HEV to humans can be mitigated by ensuring the proper cooking of game offal or by applying procedures that inactivate the virus in raw products, such as sausages. The percentages found in this study are lower than those reported in central Italy, where the consumption of raw or undercooked meat is more common [43]. A more in-depth investigation should be conducted on wild boars in Sicily, which not only has a strong hunting tradition but also involves culling plans that allow for related dietary consumption. Therefore, wild boars could potentially represent a strategic wildlife reservoir [44]. HEV cross-species infectivity has also been documented in cattle and goats [45,46,47].

The mussels and vegetables were negative for HAV, RoV and AdV, and the farmed meat and game offal were also tested for RoV and this gave a negative result. Previous studies conducted in mussels in Sicily reported occasional positivity for HAV (0.62%) and AdV (1.9%) [18,20].

*Toxoplasma gondii* was detected in different food matrices, such as farm and game meat and milk. The higher prevalence in wild boar meat suggests a significant zoonotic risk, particularly since wild game meat is often consumed with minimal processing or cooking. Most studies conducted on wild boars regarding *T. gondii* have focused on antibody detection. In studies carried out by molecular methods, the detection of *T. gondii* DNA is not uncommon. For example, surveillance of wildlife carried out in the Campania region (Southern Italy) detected *T. gondii* DNA in 17/90 (18.9%) wild boar organs [48]. A recent survey carried out in Sardinia (Italy) investigated, by nested PCR, 562 heart samples from wild boars, detecting 209 positive samples, with a prevalence of 37.2%, suggesting that wild boars could have a substantial role in transmitting the parasite to humans [49].

Indeed, wild boars have a higher risk of becoming infected with *Toxoplasma gondii* due to their natural behavior, which exposes them to oocysts in the soil while rooting and to tissue bradyzoites when scavenging. Wild boar meat is typically consumed by hunters or distributed locally (GfK, 2017). Undercooked meat consumption can amplify the risk of infection for humans, especially if associated with poor hygiene practices during handling and preparation that can lead to cross-contamination [50].

The presence of *T. gondii* in milk also poses a risk to consumers of unpasteurized dairy products. *Toxoplasma gondii* tachyzoites are the parasite stage likely to be shed in the milk of infected animals during lactation [11,51,52,53]. Pasteurization and low pH values are generally considered as sufficient to inactivate tachyzoites [11,53]. In European countries, the prevalence of *T. gondii* in raw milk samples via molecular assays has been reported to range from 4% to 11% as regards sheep milk samples [54,55] and from 4% to 65% with reference to goat milk samples [56,57,58], whereas 16% of cow milk samples were found positive [12,56]. A single sample of farmed meat was positive for *Toxoplasma gondii*, confirming a low molecular prevalence in Italy despite higher seropositivity values [59].

*Toxoplasma gondii* infections are widespread globally, with many cases remaining asymptomatic. However, *T. gondii* can lead to serious illness, particularly in congenitally infected children and individuals with compromised immune systems [60]. Foodborne *T. gondii* infections in humans may be linked to the consumption of meat, which includes cured meats and game meat [61]. Current data regarding *Toxoplasma gondii* in the food chain are limited, and existing meat inspection methods are insufficient to reduce the risk of human toxoplasmosis from meat consumption. Additionally, food safety protocols, which focus on bacterial contamination, are less effective for intracellular parasites like *T. gondii* [12].

The consumption of raw milk also emerged as a risk factor for a possible foodborne transmission route for *C. burnetii*, even if for this pathogen the risk of transmission through milk is lower than through the inhalation of aerosols from birthing materials or contact with livestock [62]. Pasteurization effectively inactivates *C. burnetii* in milk, highlighting the importance of this process in preventing milk-borne infections. While the oral route of transmission should not be disregarded, particularly for farmers producing artisanal cheese, pasteurization remains vital for ensuring consumer safety. Pregnant women, children, the elderly, and immunocompromised individuals should avoid the consumption of unpasteurized products. The increasing demand for raw milk products poses a public health risk due to the heightened potential for milk-borne diseases. Testing bulk tank milk for *C. burnetii* in sheep and goats is a relatively new non-invasive approach and can be used alongside ELISA testing to monitor herd health [63,64].

Lastly, a sample of milk and two offal meat samples from wild boars tested positive for *Leptospira* spp. The transmission of *Leptospira* spp. from animals to humans is generally as a consequence of direct excretion in urine, while transmission through the consumption of raw milk products is not a conventional route and is not frequently reported and the use of pasteurized milk for consumption and processing can significantly reduce risks [65].

On the contrary, wild boar is an important *Leptospira* spp. reservoir and can act as asymptomatic renal carriers that contribute to maintaining and sharing the infection-shedding *Leptospira* with urine in the environment [66]. The risk of transmission of *Leptospira* spp. generally comes from indirect exposure to contaminated food or water [67]. In the current study, two kidneys from wild boars were positive for *Leptospira* spp., confirming the association of this pathogen with wild boar populations, as previously reported in other studies [68,69,70,71].

Wild boar offal was found to be the food matrix most likely to harbor foodborne pathogens. Possible risk factors for wild boars also include their high population density and their opportunistic behavior, which leads them to approach urban areas in search of food. Given that wild boar meat has been authorized for consumption in Sicily, yet no official sanitary controls are performed on these products, it is crucial to recognize the potential risk of pathogen transmission associated with their consumption, especially when consumed as sausages or when undercooked.

Overall, the findings of this study highlight the significant public health risks associated with the consumption of various food products in Sicily, particularly those derived from wild game, unpasteurized milk and shellfish. Given the prevalence of pathogens such as *T. gondii*, *C. burnetii*, HEV and NoV, there is a need for enhanced food safety measures, including stricter monitoring and improved cooking and processing recommendations to reduce the risk of foodborne illnesses.

## 5. Conclusions

This study provides a comprehensive assessment of foodborne pathogens across various food matrices in Sicily, highlighting several areas of public health concern. The detection of pathogens such as *T. gondii*, *C. burnetii*, HEV and NoV in common food sources like milk, meat, mussels and game offal underscores the potential for foodborne disease transmission.

The detection of NoV in mussels indicates a significant transmission risk through raw or undercooked shellfish consumption, pointing to the importance of bivalve biomonitoring for food safety.

The high presence of *T. gondii* and HEV in wild boar offal presents a considerable risk, especially given the growing popularity of hunting, the tendency to consume wild game meat when undercooked or raw and, for HEV, the reported cross-species infectivity in cattle and goats. Official veterinary inspection activities could benefit from molecular biology analyses, which can provide valuable information on game product safety and support the epidemiological surveys on the environmental circulation of FBPs.

A relatively high detection rate for *C. burnetii* in milk was also reported, indicating a potential risk associated with consuming unpasteurized dairy products.

By analyzing these commonly consumed food products, the current study sought to contribute to the development of enhanced surveillance systems and to inform public health policies. The obtained results emphasize the need for a monitoring system for foodborne zoonotic pathogens using standardized molecular methods, alongside epidemiological studies, to improve sanitation measures and good processing and handling practices.

## Figures and Tables

**Table 1:** Details of food matrices analyzed in this study.

Food Matrix	Number	Description
Mussels	51	n. 51 hepatopancreas pool (*Mytilus galloprovincialis*)
Farmed meat	34	n. 17 beef (beef and veal)
		n. 17 pork, poultry (chicken and turkey)
Game offal	318	n. 318 offal from wild boars (spleen, liver, heart, lung, kidney, gut)
Vegetables	16	n.16 leaf salad in a bag pre-washed and ready to eat (RTE)
Milk	85	n. 37 sheep bulk milk
		n. 48 bovine bulk milk
Total	504	

**Table 2:** Details of molecular methods carried out in this study.

Pathogen	Method	Primers/Probe	Sequence 5′-3′	Molecular Target	Reference
*Toxoplasma gondii*	Real-time PCR	AF1	5′-CACAGAAGGGACAGAAGT-3′	529 bp repeat element	[21]
		AF2	‘5-TCGCCTTCATCTACAGTC-3′		
		Toxo Probe	‘5-CTCTCCTCCAAGACGGCTGG-3′		
*Coxiella burnetii*	Real-time PCR	slS1priF	‘5-CGGGTTAAGCGTGTCCAGTAT-3′	IS1111 region	[22]
		slS1priR	‘5-TCCACACGCTCCCATCACCAC-3′		
		Tqpro slS1	‘5-AGCCCACCTTAAGACTGGCTACGGTGGAT-3′		
*Leptospira* spp.	Multiplex real-time PCR	Lep-F	5′-TAGTGAACGGGATTAGATAC-3′	16S rRNA gene	[23,24]
		Lep-R	5′-GGTCTACTTAATCCGTTAGG-3′		
		Lep-Probe	FAM-5′-AATCCACGCCCTAACGTTGTCTAC-3′-BHQ1		
		LipL32-45F	‘5-AAG CAT TAC CGC TTG TGG TG-3′	LipL32	
		LipL32-286R	‘5-GAA CTC CCA TTT CAG CGA TT-3′		
		LipL32-189P	FAM-‘5-AA AGC CAG GAC AAG CGC CG-3′-BHQ1		
HAV	Real-time RT-PCR	HAV68	‘5-TCACCGCCGTTTGCCTAG-3′	5’-NCR	[19,26]
		HAV240	‘5-GAGAGCCCTGGAAGAAAG-3′		
		HAV150p	FAM-’5-CCTGAACCTGCAGGAATTAA-3′-MGB		
NoV	Real-time RT-PCR Norovirus GI	QNIF4	‘5-CGCTGGATGCGNTTCCAT-3’	ORF2	[19,27]
		NF1LCR	‘5-CCTTAGACGCCATCATCATTTAC-3’		
		NVGG1p	FAM-‘5-TGGACAGGAGAYCGCRATCT-3’-TAMRA		
	Real-time RT-PCR Norovirus GII	QNIF2	‘5-ATGTTCAGRTGGATGAGRTTCTCWGA-3’		
		COG2R	‘5-TCGACGCCATCTTCATTCACA-3’		
		QNIFs	FAM ‘5-AGCACGTGGGAGGGCGATCG-3’-TAMRA		
RoV	Real-time RT-PCR	NVP3-F Deg	‘5-ACCATCTWCACRTRACCCTC-3’	NSP3	[25]
		NVP3-R1	‘5-GGTCACATAACGCCCCTATA-3’		
		NVP3	‘5-FAM-ATGAGCACAATGTTAAAAGCTAACACTGTCAA-3’-MGB		
AdV	Nested PCR	ADE1–hexAA1885	‘5-GCCGCAGTGGTCTTACATGCACATC-3’	Ad2, Ad40, Ad41 hexon genes	[28]
		ADE2–hexAA1913	‘5-CAGCACGCCGCGGATGTCAAAGT-3’		
		ADE3–nehexAA1893	‘5-GCCACCGAGACGTACTTCAGCCTG-3’		
		ADE4–nehexAA1905	‘5-TTGTACGAGTACGCGGTATCCTCGCGGTC-3’		
HEV	Real-time RT-PCR	JVHEVF	‘5-GGTGGTTTCTGGGGTGAC-3’	ORF3	[29,30,31]
		JVHEVR	‘5-AGGGGTTGGTTGGATGAA-3’		
		JVHEVPmod	‘5-FAM-TGATTCTCAGCCCTTCGC-3’-MGB		

**Table 3:** Foodborne pathogens detected in food matrices analyzed in this study.

Food Matrix	*T. gondii* Pos/tot (%)	*C. burnetii* Pos/tot (%)	*Leptospira* spp. Pos/tot (%)	HEV Pos/tot (%)	HAV Pos/tot (%)	RoV Pos/tot (%)	AdV Pos/tot (%)	NoV Pos/tot (%)
Mussels	0/51	0/51	0/51	0/36	0/51	0/51	0/51	3/51
Farmed meat	1/34 (2.9%)	0/34	0/34	0/19	N.E.	0/19	N.E.	N.E.
Game offal	18/318 (5.7%)	0/318	2/318 (0.6%)	17/222 (7.7%)	N.E.	0/29	N.E.	N.E.
Vegetables	0/16	0/16	0/16	N.E.	0/16	0/16	0/16	0/8
Bulk milk	2/85 (2.4%)	15/85 (17.6%)	1/85 (1.2%)	N.E.	N.E.	N.E.	N.E.	N.E.
Total	21/504 (4.2%)	15/504 (3.0%)	3/504 (0.6%)	17/277 (6.1%)	0/67	0/115	0/67	3/59 (5.1%)

HEV: Hepatitis E Virus; HAV: Hepatitis A Virus; RoV: Rotavirus; AdV: Adenovirus; NoV: Norovirus; N.E.: not examined.

## Data Availability

The data are contained within the article.

## Supplementary Materials

The following supporting information can be downloaded at https://www.mdpi.com/article/10.3390/pathogens13110998/s1: Table S1: Genbank Accession Numbers of the target regions amplified in this study.
