# Supplementary material for: Foodborne Pathogens Across Different Food Matrices in Sicily (Southern Italy)
Source: Pathogens. 2024 Nov 14;13(11):998. doi: 10.3390/pathogens13110998 (PMC11597087; doi:10.3390/pathogens13110998)
Supplement: Supplementary file 1 [file pathogens-13-00998-s001.zip › pathogens-3293219-supplementary.pdf]

Table S1. Genbank Accession Numbers of the target regions amplified in this study.

Pathogen	Molecular target	Genbank Accession Number
Toxoplasma gondii	529-bp repeat element	AF146527 from 181 to 274
Coxiella burnetii	IS1111 region	NR_104916.1 from 27 to 172
Leptospira spp.	16S rRNA gene	Consensus sequences among FJ154560, FJ154600, FJ154577, FJ154571, FJ154569, FJ154568, FJ154564, FJ154563, FJ154556, FJ154555, FJ154553
	LipL32	Consensus sequences among AF121192, AF181553, AF181554, AF181555, AF181556, AF245281, AF366366, LIU89708
HAV	5′-NCR	M14707 from 68 to 241
NoV	ORF2	GI: M87661 from 5291 to 5376<br>GII: X86557 from 5012 to 5100
RoV	NSP3	X81436 from 963 to 1049
AdV	Ad2, Ad40, Ad41 hexon genes	MT277585.1 (from 18858 to 19158)
HEV	ORF3	M73218 from 5261 to 5330
